# Bladder cancer immunotherapy parallel advances in BCG optimization and next-generation vaccine platforms

**DOI:** 10.3389/fimmu.2025.1648698

**Published:** 2025-09-26

**Authors:** Fengshuo Li, Dawei Wang, Yuan Gao, Yuanshan Cui

**Affiliations:** ^1^ Department of Urology, The Affiliated Yantai Yuhuangding Hospital of Qingdao University, Yantai, China; ^2^ Department of Urology, Weifang People’s Hospital, Weifang, China; ^3^ Department of Urology, Yantai Yuhuangding Hospital, Yantai, China

**Keywords:** bladder cancer, Bacillus Calmette-Guérin vaccine, peptide-based vaccine, DC-based vaccine, virus-based vaccine, RNA-based vaccine, DNA-based vaccine, immunotherapy

## Abstract

Bladder cancer (BCa) remains a significant global health challenge with rising incidence and suboptimal outcomes in advanced stages. Although immunotherapy for urological cancers is not a new treatment, recent clinical advances have confirmed the value of immunotherapy as a urological cancer treatment. In the field of cancer immunotherapy, increasing attention has been focused on the use of cancer vaccines that activate T cells to target growing tumors. Despite Bacillus Calmette-Guérin (BCG) intravesical immunotherapy serving as the first-line treatment for non-muscle-invasive bladder cancer (NMIBC), its limitations, including systemic toxicity, BCG unresponsiveness, and rapid bladder clearance-necessitate novel therapeutic strategies. This descriptive review synthesizes recent advances in BCG optimization and emerging cancer vaccines for BCa, including peptides, antigen-presenting cells, viruses, or nucleic acids, that seeks to stimulate the patient’s immune response targeting tumor cells. Our study underscores the transformative potential of next-generation vaccines in redefining BCa management while addressing critical barriers to implementation.

## 1 Introduction

Ranking as the ninth most common malignancy globally, urothelial carcinoma (UC) represents the predominant urinary system’s cancer. It exhibits bifurcated anatomical presentations: upper tract UC and lower tract UC (1). BCa is the most common UC, ranking as the fourth most prevalent cancer in men, with an annual global incidence of >430,000 cases (2, 3). Among incident bladder cancer cases, the NMIBC accounts for three-quarters of presentations, whereas muscle-invasive and metastatic forms constitute the remaining cases (4). Although UC is more prevalent in the United States and Western Europe, its prevalence may be associated with high levels of industrialization, high smoking rates, and exposure to environmental carcinogens. In recent years, the incidence of UC in China has been increasing. Disease manifestation escalates progressively with advancing age, reaching optimal incidence thresholds in individuals aged 50 to 70 years (5, 6). The 5-year OS rate of BCa patients in China is about 72.9%, which is lower than that in developed countries (such as 80% in the United States). Therapeutic navigation for UC is hindered by significant comorbidity burdens and suboptimal patient performance metrics.

In terms of clinical treatment options, the standard treatment for NMIBC is transurethral resection of bladder tumors (TURBT) combined with postoperative intravesical infusion therapy (BCG or other chemotherapy drugs). High-risk NMIBC cohorts with BCG failure or intolerance require escalation to definitive surgical management via radical cystectomy (4). While radical cystectomy can effectively prevent local disease progression, it is associated with significant risks of postoperative complications, compromised quality of life (QoL) in survivors, and potential overtreatment of individuals whose disease might not have progressed clinically (7–9). Additionally, not all patients are eligible for cystectomy because of advanced age or severe co-morbidities (10). Because of the life-altering consequences and reduced QoL associated with radical cystectomy, many patients refuse to undergo the surgery (11). Novel therapies that have emerged, including PD-1/PD-L1 axis inhibition,which is fundamental to modern BCa immunotherapy. By preventing T-cell inactivation, anti-PD-1/PD-L1 antibodies (e.g., pembrolizumab, nivolumab, atezolizumab) reverse tumor immune evasion mechanisms. The use of these antibodies have shown promising results (12, 13). MIBC is treated by RC, neoadjuvant therapy like cisplatin-based combinations is usually used in locally advanced cases (14). For carefully identified candidates unsuitable for or declining cystectomy, bladder-preserving strategies employing maximal transurethral resection, platinum-based chemotherapy, and precision radiotherapy constitute an alternative paradigm (14). However, advanced BCa continues to demonstrate refractory progression, with contemporary therapies rarely achieving disease eradication despite therapeutic innovations.

Cancer vaccines are a promising new type of treatment. They represent a dual strategy for tumor control. Preventive vaccines (e.g., HPV vaccines) block oncogenic pathogen infections, while therapeutic vaccines activate antitumor immunity by presenting tumor antigens to T cells. Current clinical efforts are focusing on dendritic cell vaccines (e.g., sipuleucel-T for prostate cancer) and personalized neoantigen vaccines tailored to individual tumor mutations. These vaccines synergize effectively with immune checkpoint inhibitors (ICIs) by overcoming tumor immunosuppression. While sipuleucel-T remains the sole FDA/EMA-approved therapeutic cancer vaccine for metastatic prostate cancer, recent clinical efforts focus on novel platforms and combinations. BioNTech’s BNT111, an mRNA vaccine targeting melanoma antigens, has demonstrated robust T-cell activation in phase I/II trials (15). The reverse tumor immune evasion mechanisms from anti-PD-1/PD-L1 antibodies establish a permissive microenvironment for vaccine-induced immune responses,explaining the enhance efficacy observed in combination trials. Moderna and Merck’s mRNA-4157, a personalized neoantigen vaccine paired with pembrolizumab, has shown improved progression-free survival in melanoma and NSCLC (NCT03815058) (16). Preventive HPV vaccines continue to reduce cervical and oropharyngeal cancer incidence globally.

The advantages of cancer vaccines include an excellent safety profile and potential for long-term immune memory. However, challenges persist: tumor antigen heterogeneity limits universal targets; weak immunogenicity of shared antigens (e.g., MAGE-A3 in NSCLC) limits efficacy; and tumors evade immunity via Treg activation or antigen presentation downregulation. Manufacturing complexities and costs, such as cold-chain requirements and prolonged production timelines, further hinder widespread application. Ongoing trials explore novel delivery platforms (mRNA, viral vectors) and combination therapies to amplify clinical benefits. Here, we conducted a review that recalls the history, mechanisms, and limitations of adjuvant intravesical instillation of BCG, while focusing on novel vaccine therapies for BCa, aiming to provide guidance on cancer vaccine treatment in the field of urinary tract tumors.

## 2 Methods

This evidence synthesis employs a descriptive methodological framework. During April 2025, comprehensive bibliographic interrogation of PubMed and Embase databases was performed utilizing structured Boolean queries incorporating lexical variants of: (‘therapeutic vaccination’ OR ‘cancer vaccine’) AND (‘urothelial malignancy’ OR ‘bladder neoplasms’). The evidentiary corpus encompassed systematic reviews, translational investigations, and phase I-III clinical trials, with prioritization given to seminal publications. **Table 1** integrates the vaccine types and representative schemes of the new generation of bladder cancer vaccine treatment platform mentioned in this study.

**Table 1:** Next-generation vaccine platforms of bladder cancer immunotherapy.

Vaccine type		Typical strategies	Mechanism/Advantage	Limitations	Stage
Optimized BCG	Nano BCG	BCG-CS/Fe_3_O_4_-CS/GP	Prolonging the retention time of BCG in bladder	Carrier materials may cause systemic inflammation; Long term biocompatibility not verified	Preclinical (rat modal)
	Recombinate BCG	BCG-disA-OE	Overexpression of c-di-AMP activates the sting pathway and strengthens trained immunity	High production cost; Large scale production is difficult	Preclinical (mouse model)
		VPM1002BC	Knockdown of urec gene + introduction of Listeria haemolysin to promote antigen release	Clinical data were limited to small samples (n=6)	Phase II clinical trial
Peptide-Based Vaccines		S-288310	Targeting TAAs and inducing CTL responses	HLA restricted; needs to be combined with adjuvant	Phase II clinical trial
		PPV	Screening of patient specific highly reactive peptides from candidate Libraries	Only 46% of patients produce T cell responses; High production cost	Phase II clinical trial
		Suvivin-2B80-88	Targeting the inhibitor of apoptosis protein survivin	HLA-A24 positive patients only	Phase I clinical trial
Cell-based Vaccines		DC cell vaccine (WT1/HER-2/MAGE-3)	DCs loaded with tumor antigens activate T cells	Complex preparation; High production cost	Phase I/II clinical trial
Virus-based Vaccines		Nadofaragene firadenovec	Adenoviral vector mediated local expression of IFN α	Long term safety of gene therapy to be verified	FDA approval (after BCG failure)
Nucleic acid Vaccines		mRNA-4157	Individualized neoantigen mRNA vaccine	High requirements for cold chain transportation; Long individual production cycle	Phase III clinical trial
		DNA vaccines (HER-2/Flk-1)	Plasmids encode tumor antigens and activate humoral / cellular immunity	Lack of clinical data; Potential genome integration risks	Preclinical

## 3 BCG

For high-risk NMIBC management, the standard paradigm entails TURBT with subsequent adjuvant intravesical BCG administration (4). BCG originated from Albert Calmette and Camille Guérin’s early 20th century work with Mycobacterium bovis during tuberculosis vaccine development. Pioneering investigations by Zbar et al. revealed that co-administering viable neoplastic cells with BCG provoked localized inflammatory responses to BCG, effectively arresting neoplastic progression (3, 17). Pioneering work by Morales et al. in the 1970s established BCG’s therapeutic efficacy through a nine-patient cohort study, culminating in its regulatory endorsement as a primary immunotherapeutic modality for NMIBC (18).

### 3.1 Mechanisms of BCG in the treatment of bladder cancer

The exact mechanism of action behind BCG therapy has not been elucidated, but it is thought that the immunologic response destroys bladder cancer cells and prevents tumor growth with variable effects on recurrence (3, 19). While translational studies documenting BCG’s cytotoxic activity against malignant urothelium remain limited, substantial *in vitro* evidence elucidates direct BCG exposure triggers apoptosis induction or proliferative arrest in specific bladder carcinoma lineages (20). Mechanistic pathways encompass apoptosis induction, necrosis triggering, oxidative stress generation, and related cytotoxic phenomena (21).

The BCG of intravesical instillation undergoes an immune response after absorption and internalization. Following intravesical instillation, BCG adheres to bladder tumor cells through molecular docking between fibronectin attachment protein (FAP) expressed on the BCG cell wall and fibronectin (FN) present on malignant cell surfaces (21). Normal bladder epithelium features a hydrophilic, highly sulfated glycosaminoglycan (GAGs) layer carrying negative surface charges. Given the similarly anionic nature of BCG’s cell wall, bacterial adherence to healthy urothelial cells is significantly attenuated (22, 23), this selective targeting mitigates collateral injury to healthy bladder epithelium and diminishes BCG therapy-associated toxicities (21).

The internalization capacity of BCG in neoplastic versus normal urothelial lineages post-cellular uptake remains unresolved in current evidence. Following TURBT, it is an established fact that both residual bladder tumor cells and repaired normal epithelial cells can take up intravesical BCG within weeks, an essential mechanism initiating subsequent immune responses (21).

**Figure 1:**
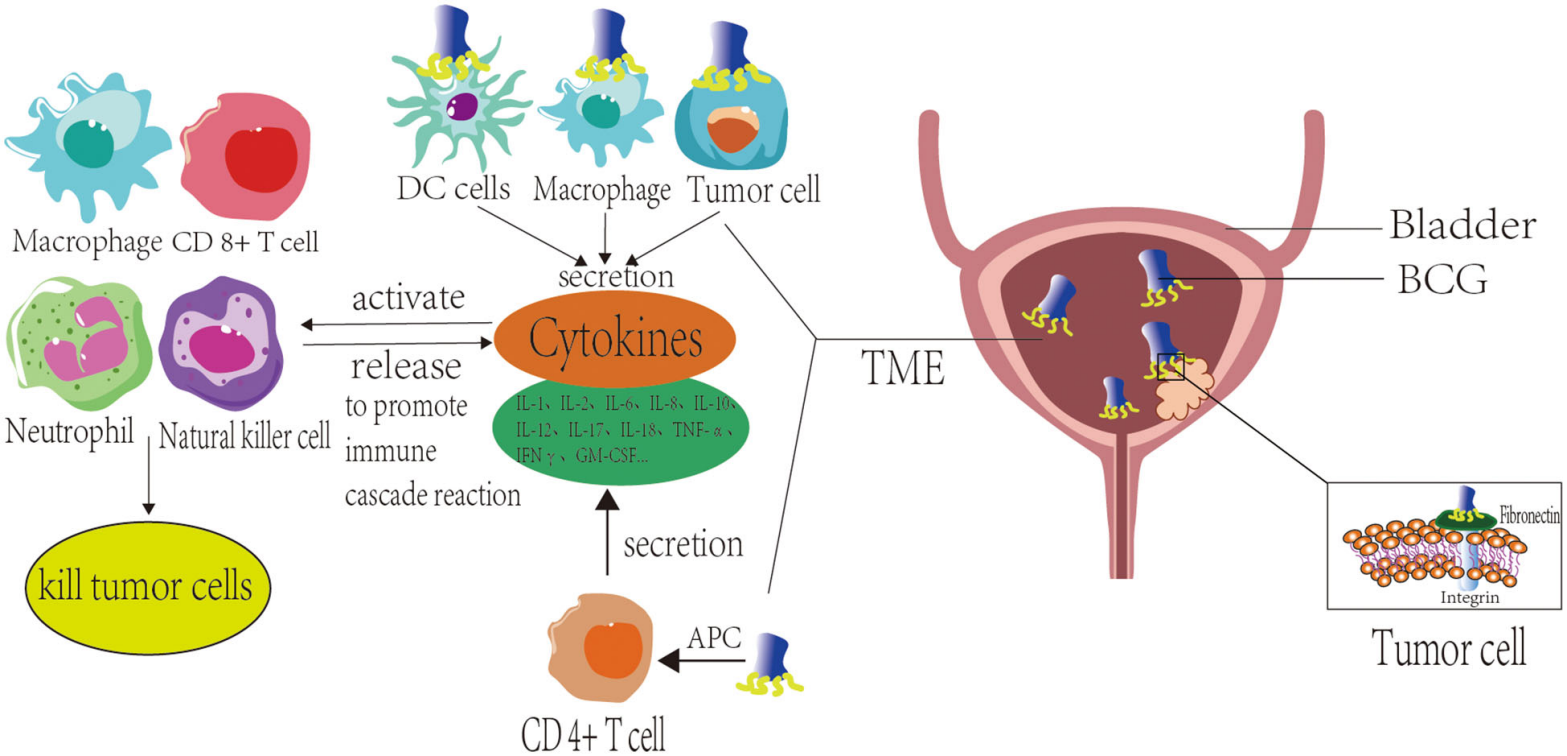
Mechanism of action of Bacillus Calmette-Guérin (BCG) vaccine for bladder cancer.

During immunological initiation, BCG serves as a pathogen-associated molecular pattern (PAMP) that engages pattern recognition receptors (PRR) on diverse cell surfaces (24, 25), BCG is internalized by macrophages or undergoes phagocytosis, leading to antigen processing by antigen-presenting cells (APCs) such as macrophages and dendritic cells. These APCs present BCG-derived antigens via major histocompatibility complex class II (MHC-II) molecules to activate CD4^+^ and CD8^+^ T lymphocytes. Concurrently, bladder tumor cells and APCs stimulate cytokine secretion, which subsequently participates in immune responses. Following antigen presentation by APCs, activated CD4^+^ T cells produce multiple cytokines, including IL-1, IL-2, IL-6, IL-8, IL-10, IL-12, IL-17, IL-18, TNF-α, IFNγ, and GM-CSF (26). BCG stimulation additionally induces tumor cells and innate immune cells to secrete substantial cytokines. These cytokines subsequently activate effector cells, including CD8^+^ cytotoxic T lymphocytes (CTLs), macrophages, neutrophils, NK cells, and others. Such activated immune cells exhibit dual functions: 1) They perpetuate cytokine release, amplifying immune cascade reactions; 2) They eliminate tumor cells through distinct mechanisms. Specifically, activated CD8^+^ CTLs exert direct cytotoxic effects on tumor cells, while macrophages significantly contribute to BCG-triggered inflammatory responses. Beyond their roles in antigen presentation as APCs and phagocytosis of malignant cells, macrophages stimulated by BCG generate macrophage secretory factors (MSFs) that induce NO production (27). Neutrophils eliminate tumor cells via phagocytosis and degranulation mechanisms. Additionally, BCG-activated neutrophils secrete neutrophil extracellular traps (NETs), which restrict BCG dissemination and reduce its clearance from the bladder (28). BCG-primed neutrophils release tumor necrosis factor-related apoptotic ligand (TRAIL) that critically mediates programmed cell death in malignant cells. Validation studies across comprehensive tumor cell panels confirm this apoptotic induction exhibits precise tumor selectivity with minimal impact on normal cellular populations (29, 30). Antibody-dependent cellular cytotoxicity (ADCC) enables NK cells to eliminate cancerous cells via antibody-mediated cytolysis (21) (**Figure 1**).

Trained immunity is one of the key mechanisms of long-term protective effect of BCG intravesical therapy for bladder cancer (31). It is a kind of “memory” ability of innate immune system: when bladder cancer cells first contact BCG, the innate immune cells (such as monocytes and macrophages) will form permanent functional reprogramming. This enables them to generate stronger inflammatory responses when they encounter unrelated stimuli such as bacterial fragments or tumor signals, thereby enhancing their anti-tumor activity. After the initial infusion of BCG, the innate immune cells of bladder wall cells are trained. BCG activates histone modifications through the NOD2/RIPK2 pathway, resulting in the enrichment of H3K4me3 (transcriptional activation marker) in the promoter region of pro-inflammatory genes (such as TNF, IL6) and a decrease in H3K9me3 (transcriptional repression marker) in the same region (32). Through metabolic reprogramming, glycolysis is enhanced, the tricarboxylic acid cycle is altered. After BCG is engulfed, autophagosomes release MDP (muroyl dipeptide) to activate NOD2 (33). During subsequent reperfusion, the trained cells showed a stronger response to residual tumor signals (such as HMGB1 and IL-1 α) in the bladder, releasing more pro-inflammatory factors such as TNF and IL-1 β, and recruiting more immune cells to kill the tumor (34). Research has found that BCG infusion can establish a systemic immune defense line. BCG activates bone marrow stem cells, generating long-lasting modified immune cells that can migrate to the bladder for sustained combat (35).

### 3.2 The limitations of BCG

Despite BCG plays an important role in the first-line therapy of NMIBC, Since 2012, the world has faced a continuous shortage of BCG production and supply. This is due to the complex production process, long cycle and the limited number of major global suppliers. The shortage of patients led to treatment delay, dose reduction or even cancellation, resulting in a significant increase in the risk of recurrence.More than 40% of NMIBC patients could not receive standard treatment due to shortage. Furthermore, BCG-induced immunotherapy can cause many side effects such as systemic infections, sepsis, and even death (3, 5). Studies show that over 70% of patients had some form of adverse impact, with 8% of patients discontinuing BCG due to toxicity (36). Intermittent urinary excretion limits instillation drug retention within the bladder, diminishing therapeutic effectiveness. For intravesical instillation therapy, efficacy directly correlates with drug concentration rather than dosage (37). That implies the duration of BCG exposure within the urinary epithelium seldom extends past the initial voiding after instillation; consequently, therapeutic instillation frequency and dosage require augmentation (38). However, a higher frequency of intravesical bladder instillation is also directly linked to specific adverse events, such as tuberculous cystitis and hematuria; these complications can detrimentally impact patients and lead to BCG therapy cessation (5, 39). This grim situation directly drives the urgent need for new alternative therapies.

## 4 Nano BCG

Therapeutic applications of nanotechnology constitute a rapidly evolving domain, where current nanomedicine delivery systems comprise magnetic nanospheres, microemulsions, and polymeric nanoparticles. The current research results (40, 41) show that nanomedicine carriers demonstrate extended drug release duration, facilitate co-loading of multiple therapeutics for combination regimens, minimize systemic toxicity, and enhance bioavailability. Given the genetic instability of bladder tumor cells and their surface overexpression of tumor antigens, BCG immunotherapy represents an ideal approach to augment BCa targeting through nanotechnology-based delivery systems (5).

### 4.1 BCG-chitosan

CS is a cationic polymer exhibiting strong bioadhesion, demonstrates potent permeation-enhancing properties (41), which facilitates therapeutic penetration across the urinary epithelium and enhances drug dispersion depth within the bladder wall during intravesical administration (5). When utilized in intravesical instillation therapy, CS additionally promotes immune activation. In a rodent bladder cancer model, Erdoğar et al. investigated the antitumor efficacy of BCG-loaded CS nanomaterials. Tumor-bearing rodents receiving BCG-CS exhibited substantially prolonged survival compared to the BCG-only cohort, with histopathological analyses verifying BCG’s antitumor action across all treatment arms. Furthermore, pronounced nanoparticle accumulation in bladder tissues of the BCG-CS group indicated potential induction of robust immune responses by BCG. CS significantly augmented BCG internalization, with the BCG-CS formulation markedly enhancing therapeutic outcomes in bladder tumor immunotherapy (5, 42).

Another viable solution for extending BCG’s therapeutic duration within the bladder wall involves temperature-sensitive hydrogels constituted by CS and glycerophosphate (GP). At room temperature, CS/GP aqueous solutions remain low-viscosity fluids, transforming into viscous gels at physiological temperature. Due to this characteristic, CS/GP can reserve BCG and release it slowly (43). Antiinflammatory drugs delivered via this system in a rat model of interstitial cystitis confirm its therapeutic benefits (5, 37).

The superparamagnetic nature of Fe_3_O_4_ magnetic nanoparticles (an iron oxide type) enables retention of the thermosensitive hydrogel during bladder voiding and promotes adherence to the bladder wall (44). The combination of Fe3O4 magnetic nanoparticles and CS/GP will solve the significant limitation of the brief duration instillation drugs remain within the bladder. A temperaturesensitive hydrogel composed of CS and GP combined with Fe3O4 magnetic nanoparticles to form an *in situ* gel system (Fe3O4-CS/GP) was proposed, it transitions to a viscous hydrogel under physiological conditions, while its magnetic injectable form substantially extends drug persistence within bladder tissue when subjected to magnetic guidance. Applying this characteristic, Zhang et al. incorporated BCG to this system (Fe3O4-BCG-CS/GP), discovered that BCG’s controlled release in the bladder was enabled by the hydrogel slow-delivery mechanism, and the antitumor and local immunostimulatory activity of Fe3O4-BCG-CS/G was better than BCG alone *in situ* BCa rat model. Immunohistochemical analysis revealed enhanced submucosal CD4+ lymphocyte infiltration in rat bladders treated with Fe_3_O_4_-BCG-CS/GP. Furthermore, this composite significantly elevated cytokine production (notably IL-2 and IFN-γ) relative to BCG monotherapy (45, 46).

### 4.2 BCG-cell wall skeleton

Serious side effects due to intravesical BCG infusion, such as systemic infections and sepsis, it is essential to development of non-infectious, low-toxicity BCG immunotherapies. CWS is extracted from the cell wall components of Nocardia rubra, and has the effect of enhancing immune function, stimulating immune cells, and eliminating inflammation. These features make BCG-CWS the primary center of activity for BCG (47). The limited clinical adoption of BCG-CWS stems from three key constraints: (1) Proneness to aggregation impedes development of stable aqueous formulations; (2) Inefficient cellular internalization by malignancies; (3) Dependency on oil/water (O/W) emulsion detergents for mechanical dispersion *in vivo*, which triggers severe inflammatory sequelae (48). Thus, effective utilization of BCG-CWS in O/W emulsions for BCa therapeutics remains profoundly challenging (5).

To address limitations of BCG-CWS O/W therapies, Nakamura’s team developed oil/detergent-free nanoparticles (CWS-NP/LEEL) via the liposome evaporation emulsified lipid (LEEL) technique (40). They observed that vitro analyses demonstrated efficient internalization of CWS-NP/LEEL by MBT-2 murine bladder tumor cells, resulting in tumor growth suppression with minimal bladder tissue impact. This intervention substantially prolonged median murine survival duration. In subsequent trials, by using naive CD4 T cells isolated from four healthy human donors, Nakamura’s team demonstrated CWS-NP/LEEL’s capacity to polarize the Th1/Th2 balance toward Th1 immunity. BCG-CWS-NP/LEEL has the potential to be a non-toxic treatment alternative to BCG alone and mitigate disease burden of patients with BCa.

## 5 Recombinant BCG

rBCG refers to a type of vaccine developed through genetic engineering techniques to introduce targeted modifications (such as gene knockout, insertion, or replacement) into the genome of the traditional BCG vaccine. These modifications aim to enhance its immunogenicity, improve safety, or introduce new functional properties. The core characteristic of rBCG lies in the deliberate alteration of BCG’s genetic material to achieve specific therapeutic or preventive goals. These rBCG strains are a mechanistic approach to efficacy enhancement of BCa treatment.

### 5.1 Exploration of rBCG-fibrin D-mannose specific adhesin

As a naturally produced adhesion protein, FimH serves as a tumor adjuvant (49). It is a bacterial adhesin protein; FimH-mediated adhesion to mannose residues on urinary tract epithelial surfaces underpins *Escherichia coli* urinary infections. This happens to be used to address the low attachment of BCG treatment; improving the retention of BCG to the uroepithelium may enhance its tumoricidal effects. FimH can promote dendritic cell (DC) activation by upregulating the expression of co-stimulatory molecules and major histocompatibility complex class II (MHC-II). Zhang et al. (2022) successfully overexpressed FimH on the surface of BCG, resulting in rBCG-urinary epithelial cell adhesion, longer retention time in the bladder uroepithelium, and faster internalization of rBCG-surface FimH(rBCG-S. FimH) by uroepithelial cells. Furthermore, in murine bladder tumor models and PBMC-treated human BCa lines rBCG-S. FimH potentiated DC antigen presentation through TLR4-MyD88-pJNK-NF-κB pathway activation, corroborating its immunostimulatory function (50). BCG’s immunological capabilities undergo further enhancement through recombinant methodologies.

### 5.2 Exploration of rBCG-streptococcal of complement

When BCG acts on the urothelium of the bladder, it triggers non-specific immunity. Urothelial cells kill BCG by secreting antimicrobial peptides (AMPs), which activate the host immune defense system. Other types of cells avoid AMPs by secreting alternative specific proteins like SIC and D-alanyl carrier protein ligase (dltA) (51). Choi et al. (2015) speculated that the poor efficacy of BCG treatment in patients with BCa could be due to the killing of BCG by AMPs, resulting in reduced BCG internalization (52). Kim and Seo next engineered a recombinant BCG strain producing SIC, examining its capacity to resist antimicrobial peptides while inducing targeted immune responses (53). Their findings demonstrated rBCG-SIC was more resistant to AMPs. Infection of BCa cells with this construct provoked markedly elevated IL-6 production and substantially suppressed T24 cell viability relative to conventional BCG. Within an *in situ* murine BCa model, tumor volumes measured following 10-day treatment were further documented as significantly reduced in the rBCG-SIC cohort versus the BCG group. These collective findings position rBCG-SIC as a promising therapeutic alternative for BCG-unresponsive NMIBC (53).

### 5.3 Exploration of BCG and cytokine mixture

Previous clinical trials have shown that the combination of BCG and cytokines can be used in different stages of tumor treatment (54). Cytokine-secretable rBCG has the potential to improve the therapeutic effect of immunotherapy for BCa.

Cutting-edge research in this field demonstrates that genetic engineering significantly enhances the immunostimulatory properties of BCG. Yamada et al. (2000) constructed a rBCG by ligating the IL-2 gene with a Mycobacterium tuberculosis antigen gene using the plasmid vector pS0246. Experimental data revealed that this engineered strain continuously secretes biologically active IL-2, exhibiting markedly superior cytotoxicity against MBT-2 bladder cancer cells compared to conventional BCG. Notably, while exogenous IL-2 supplementation alone could augment BCG efficacy, endogenously expressed IL-2 by rBCG demonstrated up to 100-fold greater potency at equivalent concentrations (55). In subsequent mechanistic studies, Luo et al. (2004) engineered BCG to autonomously express interleukin-18 (IL-18), revealing that this recombinant strain robustly activated Th1-polarized immune responses and significantly enhanced macrophage-mediated clearance of MBT-2 tumor cells. Comparative analyses indicated that both cytokine-expressing rBCG variants overcame the therapeutic limitations of conventional BCG by synergistically modulating innate and adaptive immunity (56). Collectively, these findings highlight that genetically modified rBCG leverages sustained endogenous cytokine release to amplify localized immune activation within the bladder microenvironment while optimizing therapeutic dosing. The reduced bacterial load required to achieve equivalent antitumor effects presents a promising strategy to mitigate dose-dependent adverse effects, such as local irritative symptoms and systemic toxicity, commonly associated with intravesical BCG therapy. This innovative approach not only improves clinical efficacy in BCa immunotherapy but also establishes a dual advantage of enhanced safety and precision in treatment delivery.

Interleukin-15 (IL-15), a pro-inflammatory cytokine with demonstrated antitumor potential, has been extensively studied as a therapeutic agent in oncology (57, 58). Beyond its critical role in sustaining NK cells and memory CD8+ T cells, IL-15 regulates neutrophil activation and migratory behavior, positioning it as a key modulator of innate and adaptive immunity (59, 60). Building on this mechanistic foundation, Takeuchi et al. (2016) engineered a recombinant BCG strain co-expressing IL-15 and the Ag85B fusion protein (BCG-IL-15). In tumor-bearing mouse models, BCG-IL-15 significantly extended survival outcomes, correlating with elevated neutrophil infiltration and heightened chemokine levels—specifically macrophage inflammatory protein (MIP) -2 and MIP-1α—in bladder tissues (61). This enhanced activity may be attributed to IL-15’s capacity to orchestrate neutrophil trafficking during inflammatory responses. Studies by Verri et al. (2007) revealed that IL-15 initiates signaling cascades driving MIP-2 and MIP-1α production, which in turn amplify neutrophil chemotaxis. Such immunomodulatory mechanisms likely underpin the superior antitumor efficacy of BCG-IL-15 compared to conventional BCG, as the cytokine potentiates both direct neutrophil-mediated cytotoxicity and microenvironmental immune activation (62).

### 5.4 Re-engineered BCG overexpressing cyclic di-AMP

BCG-disA-OE constitutes a recombinant BCG variant generated through fusion of the endogenous diadenylate cyclase gene disA to a high-activity promoter. This genetic modification drives 300-fold upregulation of disA expression and elevates cyclic-di-AMP yields by 15-fold (63). Compared with wild-type BCG, BCG-disA-OE significantly increased STING pathway activation in macrophages as measured by IRF3 induction. Alok Kumar Singh et al. (2022) generated BCG-disA-OE and evaluated its assessment of cytokine induction capacity in human monocyte-derived macrophages (HMDMs), primary murine bone-marrow-derived macrophages (BMDM), dendritic cells (BMDC), and a macrophage cell line revealed consistently elevated expression of IRF3, IFN-β, TNF-α, and IL-6 across all myeloid populations following BCG-disA-OE stimulation. This response substantially exceeded levels observed in wild-type-BCG-exposed cells. Notably, enhanced cytokine production persisted in IFN-γ-primed human MDM and murine BMDM cultures. That means BCG-disA-OE exhibits enhanced potency in stimulating pro-inflammatory cytokine production across primary human MDM, murine primary macrophages, and macrophage cell lines compared to wild-type BCG (64).

Immunity driven by BCG involves macrophage polarization toward pro-inflammatory phenotypes. Flow cytometry analysis of murine and human primary macrophages exposed to BCG-disA-OE (overexpressing cyclic-di-AMP) versus wild-type BCG revealed distinct immunomodulatory effects. Using both mouse and human primary macrophage ex vivo models, Alok Kumar Singh et al. (2022) found that, relative to wild-type BCG, BCG-disA-OE induces enhanced macrophage activation toward inflammatory polarization while simultaneously suppressing the development of immunosuppressive cell populations, including M-MDSCs.

Research indicates cyclic dinucleotides mediate the recruitment of inflammatory macrophages exhibiting heightened phagocytic activity (65–68). In human monocyte-derived macrophages (HMDMs), BCG-disA-OE exposure significantly increased phagocytosis of IgG-opsonized FITC-latex beads by macrophages exposed compared to wild-type BCG. Furthermore, BCG-disA-OE exhibited greater co-localization with autophagy markers LC3B and p62 in IFN-γ-activated macrophages, indicating robust STING pathway-driven autophagic processing. In contrast, wild-type BCG showed minimal autophagy induction. These findings demonstrate BCG-disA-OE substantially exceeds wild-type BCG in elevating macrophage phagocytic activity and autophagic flux. This enhancement mechanistically links to improved peptide antigen presentation via MHC class-II molecules (69, 70).

By quantifying the activation histone methylation marker H3K4me3 present in the TNF-α and IL-6 promoters, using a chromatin immunoprecipitation polymerase chain reaction (ChIPPCR) assay, Alok Kumar Singh et al. observed that BCG-disA-OE exposure demonstrated superior enrichment of this epigenetic mark versus wild-type BCG. These data establish that cyclic-di-AMP-overexpressing BCG elicits substantially heightened epigenetic reprogramming hallmark of trained immunity characteristic of trained immunity (64). They also found that compared with wild-type BCG, BCG-disA-OE provoked a metabolic rewiring consistent with trained immunity characterized by diminished kynurenine buildup alongside elevated glycolytic intermediates, NOS-derived compounds, and itaconate synthesis. Because of these characteristics, BCG-disA-OE exhibits enhanced therapeutic efficacy in experimental animal models of immunotherapy.

In terms of security, BCG-disA-OE demonstrates improved safety profiles compared to wild-type BCG in murine models despite stronger pro-inflammatory responses. Alok Kumar Singh et al. evaluated safety in two separate mouse models. They used an immunocompetent BALB/c mouse model of aerosol exposure and measured the lung bacillary burden after 4 weeks, when adaptive immune responses are maximal. Quantification revealed 0.43 log10 fewer colony-forming units of BCG-disA-OE versus wild-type BCG in murine lungs, indicating reduced proliferative capacity. Subsequent low-dose aerosol challenge demonstrated statistically significant survival enhancement in BCG-disA-OE-treated cohorts compared to wild-type BCG controls (64).

### 5.5 VPM1002BC (DeltaureC hly+rBCG)

VPM1002BC constitutes the exclusive clinically implemented rBCG therapy, utilizing a viable recombinant Mycobacterium bovis strain. Based on the traditional BCG, VPM1002BC is modified by the following means of genetic engineering: the urease C gene is knocked out, and the *hly* gene of *Listeria monocytogenes* is introduced into VPM1002BC. On the one hand, it weakens the ability of bacteria to survive in the host cell, and on the other hand, it helps antigens escape to the host cytoplasm and activate a stronger immune response. Rentsch et al. pioneered clinical documentation of VPM1002BC administration in six BCG-unresponsive NMIBC cases, reporting therapeutic outcomes and induced immune modifications. Biospecimen analysis (urine/whole blood) revealed substantially elevated plasma TNF and Th1 cytokine concentrations post-intervention. Furthermore, antigenic challenge with Mtb-PPD triggered significantly heightened GM-CSF and IFN expression within blood-derived CD4^+^ T lymphocytes. All subjects completed the full six-course intravesical instillation regimen (5, 71).

## 6 Peptide-based vaccines

Cancer vaccination efficacy fundamentally relies on T lymphocytes’ specific identification of tumor antigens. Ideal TAAs demonstrate differential overexpression in malignant versus healthy tissues and exhibit high binding affinity to human leukocyte antigen (HLA), facilitating efficient presentation to T cells (72). Tumor antigens are classified in tumor-associated antigen (TAA) and tumorspecific antigen (TSA).

Peptide-based vaccines can utilize TAAs or TSAs. Early designs focused on short peptide constructs (<15 amino acids) that directly bound MHC class I molecules to activate CD8+ T cell responses (73). However, these minimal epitopes frequently failed to induce robust antitumor immunity due to insufficient costimulatory signals, often resulting in immune tolerance rather than protective responses (74). This limitation prompted the development of synthetic long peptides (SLPs), typically consisting of approximately 30 amino acids. Unlike their shorter counterparts, SLPs require processing by APCs before epitope presentation, enabling simultaneous loading onto both MHC class I and II molecules. This dual presentation facilitates coordinated CD8+ cytotoxic T cell and CD4+ helper T cell activation, theoretically enhancing immune efficacy (73). Despite demonstrating immunological activity in preclinical models, clinical trials of peptide vaccines have shown limited therapeutic success, a phenomenon potentially attributable to the MHC-restricted nature of epitope recognition and subsequent limitations in population-wide applicability (75).

To address these limitations, multiple strategies have been explored to enhance peptide vaccine efficacy. Preclinical studies suggest that conjugating peptides to carrier proteins, such as keyhole limpet hemocyanin (KLH) or heat shock proteins (HSPs), can amplify APC activation by improving antigen uptake and processing (76). Additionally, platforms like virus-like particles (VLPs) or bacterial vectors have shown promise due to their intrinsic ability to activate APCs and facilitate targeted antigen delivery to lymph nodes (77, 78). Structural modifications of peptides, including the incorporation of CTL epitopes alongside T helper epitopes, may further synergize CD8+ and CD4+ T cell responses for broader immune activation (79). Finally, adjuvants—immune-stimulating agents—have been integrated to bolster leukocyte recruitment, proliferation, and effector function, as well as to promote their migration to tumor microenvironments (TME) (80). Peptide-based vaccines offer multifaceted advantages including straightforward design, minimal contamination risks, and low adverse event potential; however, their immune potency is constrained by HLA restriction and obligatory adjuvant requirements (75, 77, 80, 81). Multiple clinical trials in patients with UC treated with peptide-based vaccines have shown promising efficacy, including enhanced survival rates and reduced rates of tumor recurrence. This section summarizes key clinical studies highlighting these outcomes.

### 6.1 S-288310

S-288310 represents a subcutaneously administered peptide vaccine integrating two tumor-associated antigens: DEPDC1 and MPHOSPH1. These targets were identified via expression profiling and individually validated for immunological activity in UC patients. In a phase I/II clinical trial involving 32 patients with UC, the peptide-based vaccine S-288310 exhibited both safety and immunogenicity, successfully inducing peptide-specific CTL responses. The study reported a disease control rate (DCR) of 56.3% and a 2-year OS rate of 32% (82). A nonrandomized phase II trial evaluating the combination of a subcutaneously administered MPHOSPH1/DEPDC1 multi-peptide vaccine and intravesical BCG therapy reported significant clinical and immunological outcomes, including a 2-year relapse-free survival (RFS) rate of 74% and peptide-specific CTL response rates ranging from 75.8% to 77.5% (83). These robust findings, characterized by durable efficacy over a 2-year period, highlight the need for expanded clinical validation through controlled, large-scale studies.

### 6.2 Personalized peptide vaccination

PPV is another vaccination modality based on subcutaneous use of the most reactive peptide selected from a large number of candidate antigens (84).The corresponding peptide is usually selected in a predefined peptide pool based on the serological HLA typing of patients (HLA-A24 or HLA-A2 positive patients). Then the peptide vaccines were selected by monitoring the pre-existing immune response of patients to peptides (such as CTL precursors and lgG levels). The immunological basis of PPV is that the peptides selected by personalization can induce HLA restricted tumor specific cytotoxic T cell (CTL) activity, and are expressed in a variety of tumor cell lines, supporting its potential role in immunotherapy. A Phase I trial evaluated the personalized peptide vaccine (PPV) in 10 pretreated UC patients, all patients were histologically confirmed as locally advance (≥T3,N1) or metastatic (M1) UC. ALL of them underwent MVAC chemotherapy (methotrexate, vinblastine, adriamycin, cisplatin) but the patients did not respond to it or disease progression. The study reported a best overall response of one complete response (CR), one partial response (PR), and two cases of stable disease (SD), alongside six instances of progressive disease (PD). The cohort exhibited a median OS of 8.9 months, with responders demonstrating durable clinical benefit—notably, the duration of response reached 24 months in these patients (84). In a randomized phase II trial involving 80 cisplatin-refractory UC patients, PPV underwent comparison against standard supportive care. Although failing to demonstrate therapeutic advantage for progression-free survival (PFS), the vaccine exhibited favorable safety and extended OS with a hazard ratio (HR) of 0.58 (p = .049) (85). A subsequent phase II monotherapy trial evaluated the PPV approach in 48 previously treated metastatic upper tract urothelial cancer (UTUC) patients. PPV was well tolerated without severe AEs, and induced a specific T-cells response in 46% of the patients, related to a longer OS with respect to patients with negative response (HR 0.37; 95%CI, 0.16–0.85; p = .019). While median OS reached 4.5 months, combination therapy with PPV plus chemotherapy extended this duration to 13 months in the subgroup cohort (representing 60% of participants) (86). PPV remains one of the most promising peptide-based vaccine strategies, showing extraordinary persistence, while the clinical benefit is confined to only a subset of patients. Further research is required to identify biomarkers or clinical characteristics that define patient subpopulations most likely to derive significant therapeutic benefit from PPV.

### 6.3 Survivin-2B80-88

Survivin (BIRC5), as an inhibitor of apoptosis protein, is highly expressed during embryonic development and almost absent in normal adult tissues. It is abnormally high expressed in most malignant tumors (including urotheial carcinoma), and is closely related to tumor proliferation, invasion, drug resistance and poor prognosis, so it is regarded as a valuable tumor associated antigen target. Survivin-2B80-88, characterized as an HLA-A24-restricted antigenic apoptosis inhibitor and CTL target, underwent phase I safety and activity validation (87). Subsequently, a study divided 30 patients with HLA-A24 positive/surgically resected primary tumor but failed to receive chemotherapy into two groups according to Survivin-2B80–88 vaccination and incomplete Freund’s adjuvant (IFA) or receiving the same vaccine and IFA but additionally combined with IFNα. The survival of the two groups was compared with that of the control group composed of 18 patients with UC who did not receive vaccine treatment. The results showed that the optimized vaccine scheme with IFNα did not significantly enhance the effect, and compared with control group, the experimental group was associated with prolonged overall survival.(p = .0009) (88). Further studies are probable, optimally validating these outcomes. However, HLA-A24 restriction means that the vaccine is theoretically only applicable to patient expressing HLA-A24 alleles.

### 6.4 PGV_001

Recent advances yielded a personalized peptide genome vaccine (PGV_001) for subcutaneous/intradermal delivery, The included peptides were derived from genomic mutations unique to the patient’s tumor. The vaccine platform analyzes tumor genome/transcriptome data to predict individualized neoantigens by applying the openvax framework (89). Across diverse solid tumors including UC, monotherapy and atezolizumab combination regimens underwent safety/efficacy assessment in adjuvant/metastatic contexts, demonstrated the ability to induce neoantigen-specific immune reactivity (90, 91).These preliminary results indicate that mRNA based personalized neoantigen vaccine platforms have the potential to induce immune responses in UC and warrant further research.

### 6.5 Combination of peptide-based vaccines and BCG

Combination strategies integrating peptide-based vaccines with BCG immunotherapy have yielded promising outcomes. For instance, a regimen combining an intradermal NY-ESO-1 peptide vaccine, GM-CSF as an adjuvant, and BCG demonstrated a favorable safety profile and immunogenicity in a small cohort of 6 UC patients, successfully inducing antigen-specific antibody production and a dominant CD4+ T-cell-mediated immune response (92). A nonrandomized Phase I open-label exploratory trial evaluated a recombinant MAGE-A3 protein vaccine combined with the immunostimulant AS15, administered either alone or alongside BCG intravesical therapy, in 24 patients with NMIBC. The regimen demonstrated safety and elicited detectable vaccine-specific T-cell responses in 50% of patients, though clinical efficacy endpoints such as tumor response were not assessed in this preliminary study (93). Regrettably, the MAGNOLIA phase II study—designed to evaluate adjuvant recombinant MAGE-A3/AS15 activity post-RC for MIBC—was discontinued early due to futility observed in concurrent phase III trials across alternative malignancies (94).

### 6.6 CDX-1307

An intradermal peptide vaccine, engineered by fusing a human monoclonal antibody targeting the mannose receptor with the tumor-associated antigen human chorionic gonadotropin beta subunit (β-HCG), demonstrated a favorable safety profile in a Phase I trial. However, the subsequent Phase II trial evaluating this vaccine in UC patients was terminated prematurely due to insufficient patient enrollment, highlighting challenges in clinical translation despite early promise (95).

## 7 Cell-based vaccines

Cell-based vaccine platforms constitute an antigen delivery methodology harnessing either malignant cells or immune cells sourced from patients, both primed with TAAs. This therapeutic paradigm has investigated diverse configurations—encompassing intact cellular preparations and subcellular components—exemplified by neoplastic cells alongside engineered autologous oncologic or immunologic units. Vaccines deploying tumor cells operate through delivery of inactivated oncologic material to provoke immunity against numerous TAAs. Nevertheless, their clinical utility remains compromised by suboptimal immunostimulatory capacity, presumably due to coexisting immunosuppressive mediators (96). Various ways of improving it have been tried, facilitating cell death, such as incorporating IFN gene-activating nanoparticles or employing delivery systems like emulsions, liposomes, or polymeric particles (97). Additionally, tumor cells can be genetically modified to express co-stimulatory molecules (e.g., IL-21, IL-7) or receptors such as chemokine receptor-7, aiming to improve lymphoid homing, antigen presentation, and the potency of immune activation (98, 99).

Immune cell vaccines typically rely on DC-based approaches. Patient-derived monocytes and hematopoietic stem cells are sourced from either peripheral or umbilical cord blood specimens, loaded with TAAs, and administered via intravenous, subcutaneous, or intradermal routes. Antigen loading strategies include direct pulsing (100), mRNA electroporation (101), viral transduction, fusion with tumor cells (102), or incubation with tumor lysate (103). DC vaccines directly present TAAs to T cells and may facilitate antigen transfer to endogenous cross-presenting DCs (104). Their effectiveness is influenced by the DC subtype: conventional DCs and myeloid/plasmacytoid DCs engage distinct T-cell activation pathways (105), and combining these subsets could enhance vaccine immunogenicity (106).

The route of DC administration may affect the immune response type due to interactions between DCs and diverse T-cell subsets in lymph nodes across anatomical sites, and combining multiple administration routes could amplify immune activation (107). To enhance DC vaccine efficacy, various adjuvants have been investigated, with commonly studied candidates including IL-2 (108), GM-CSF (95), IL-4-secreting autologous fibroblasts (109), and KLH (110). An emerging approach in DC vaccination is *in situ* vaccination, which aims to boost intratumoral DC infiltration and activation by inducing localized antigen release. This strategy involves injecting FMS-like tyrosine kinase 3 ligand (to recruit DCs) and a TLR agonist (to activate DCs) directly into the tumor, combined with localized irradiation to trigger TAAs release. A phase I trial in lymphoma patients demonstrated promising anti-tumor T-cell responses and tumor regression (111). Among diverse cancer vaccines, DC-based formulations currently demonstrate superior efficacy. These regimens exhibit safety profiles and elicit potent immune activation; however, clinical application remains constrained by intricate, costly production demands necessitating patient-specific manufacturing (112). Subsequently outlined are highly encouraging investigational studies pertaining to cell-based cancer vaccines.

### 7.1 DCs-based vaccine loaded with WT1

A phase I-II trial evaluated a DC-based vaccine loaded with Wilms’ tumor 1 (WT1) combined with the adjuvant OK432—a penicillin-inactivated, lyophilized preparation of Streptococcus pyogenes that enhances DC activity via TLR-4 signaling. Patients with advanced UC (n=2), NMIBC (n=3), or RCC (n=5) received the adjuvant and intradermal vaccine alongside standard therapies (chemotherapy or TKIs). The regimen was well-tolerated and showed clinical activity: 70% of RCC patients achieved SD, no advanced UC patients benefited, while all three NMIBC patients attained prolonged RFS (26.8, 84.8, and 33.4 months post-DC initiation), with two avoiding cystectomy. Despite the small cohort, these results highlight the promise for NMIBC, warranting validation in larger trials (100).

### 7.2 DC-based vaccine loaded with HER-2

An intradermal DC-based vaccine carrying human epidermal growth factor receptor 2 (HER-2) was engineered. DCs underwent adenoviral modification for HER-2 targeting (113). A phase I trial enrolled 21 HER-2-expressing advanced UC patients with prior therapy. Initial findings demonstrated acceptable safety and encouraging efficacy, evidenced by a 33.3% clinical benefit rate (114).

### 7.3 DC-vaccine loaded with MAGE-3

Preclinical models (115) and a phase I/II trial involving four advanced UC patients assessed a MAGE-3-loaded subcutaneous DC vaccine. Outcomes documented three partial responses (PR) with no major toxicities observed (116). Currently, no further trials are active to our knowledge; ideally, future investigations may substantiate these findings.

## 8 Virus-based vaccines

The virus-based vaccine utilizes a virus whose encoded genes have been altered to incorporate TAA sequences. These viral vectors demonstrate high efficiency in infecting cells and replicating within the cytoplasm. They can effectively deliver substantial quantities of recombinant genetic material while maintaining their infectious capability, and possess the potential to stimulate both cellular immunity and humoral immune responses (117). Furthermore, oncolytic viruses mediate direct tumor lysis, thereby stimulating antigen and viral particle liberation (118).

The virus can be administered in either live attenuated or inactivated forms, though virus-like particles (VLP) are more commonly employed (78). The predominant viral species utilized include adenovirus (119), poxvirus (120), HSV (121), measles (122), reovirus (123), and coxsackievirus (124). Notably, adenovirus is regarded as one of the most effective vectors due to its stability, broad tissue tropism, ease of production, and the adaptability of its genetic material (124). Similarly, poxviruses exhibit the capacity to accommodate large antigenic payloads and demonstrate potent immunogenicity mediated by both TLR-dependent and TLR-independent cytokine pathways (125). To minimize replication in normal cells, the viruses are typically genetically modified to selectively target tumor cells (126).

A significant challenge of virus-based vaccines lies in the host’s natural immune response, which can neutralize the viral vector. To circumvent this issue, sequential administration of distinct viral vectors expressing the same tumor antigen has been proposed (126). Furthermore, inhibitory pathways in the TME may restrict efficacy, prompting the development of diverse strategies. Primarily, combining the vaccine with ICI (127), RT (128), or chemotherapy (129) has demonstrated encouraging outcomes. Secondarily, encouraging strategies encompass targeting immunosuppressive molecules like yes-associated protein—a transcriptional coactivator fostering immunosuppressive milieus generated by Tregs (130)—or TGF-β (131). Finally, the virus’s genetic code can be engineered to incorporate immune-regulating genes like human mucin-1, IL-12, and GM-CSF (119). Below, we summarize key findings from clinical trials evaluating virus-based vaccines in UC patients.

### 8.1 CG0070

The adenovirus-based vaccine CG0070, engineered to express GM-CSF, was developed as an oncolytic virus selectively replicating in retinoblastoma-deficient UC cells (119). The transcription of GM-CSF is regulated by the E2F-1 promoter, which is specifically active in UC cells exhibiting retinoblastoma pathway defects, ensuring tumor-selective GM-CSF production. In the phase I BOND trial, intravesical administration of CG0070 demonstrated safety and efficacy, achieving a CR rate of 48.6% (119). The subsequent phase II BONDII trial evaluated intravesical CG0070 following pretreatment with dodecyl maltoside (a transduction-enhancing agent) in 45 patients with BCG-relapsed NMIBC. At the 6-month mark, CR incidence reached 47% in the entire cohort, rising to 58% among pure carcinoma *in situ* (CIS) cases and declining to 33% for pure Ta/T1 presentations and 50% in those with mixed presentations (132). These promising outcomes prompted a phase II trial investigating CG0070 combined with pembrolizumab in NMIBC patients, the combination synergizes by overcoming PD-1/PD-L1-mediated exhaustion,potentiating vaccine induced immune responses, which reported preliminary durable CR rates of 87.5% (133). This synergistic approach continues to be explored in ongoing clinical trials.

### 8.2 CVA21-based vaccine

A phase I CANON trial evaluated an intercellular adhesion molecule-1 (ICAM-1)-directed vaccine built on Coxsackievirus A21 (CVA21) in 15 NMIBC patients. Preoperative intravesical CVA21 delivery was implemented as monotherapy or in combination with mitomycin-C, demonstrating good tolerability and activity. One CR was observed, with tissue biopsies revealing significant inflammatory changes indicative of an “immunologically hot” TME (124). In a separate phase I-II trial, intravenous CVA21 combined with pembrolizumab was tested in metastatic NSCLC and UC patients. Among 43 UC patients, the objective response rate (ORR) was 20%, comparable to pembrolizumab monotherapy outcomes (134). Due to this lack of enhanced efficacy, the strategy was not pursued further in UC populations.

### 8.3 Dryvax vaccinia virus

A phase I trial investigated intravesical neoadjuvant Dryvax vaccinia virus administration in four NMIBC patients before radical cystectomy (RC). The treatment, inspired by prior positive vaccinia virus vaccination outcomes in melanoma metastases (81), resulted in no severe systemic adverse events. Histological analysis revealed substantial mucosal/submucosal inflammatory infiltration and confirmed viral infection. At a median follow-up of 4 years, three of four patients remained recurrence-free (135). While these preliminary findings are encouraging, larger-scale trials will be required to validate the vaccine’s therapeutic potential.

### 8.4 GM-CSF/TRICOM-encoding fowlpox (FPV) viral vector-based recombinant poxvirus vaccine

TRICOM, previously evaluated in multiple trials, represents a multi-molecule immunostimulatory approach aimed at enhancing antitumor immunity. A recombinant poxvirus vaccine using the fowlpox (FPV) viral vector, encoding either GM-CSF or TRICOM (a molecular triad comprising three immune costimulatory constituents: B7.1, ICAM-1, and leukocyte function-associated antigen-3 [LFA-3]), was intravesically administered to 20 BCG-unresponsive NMIBC patients scheduled for surgery. Histological evaluation of cystectomy specimens confirmed effective viral infection/transfection. The treatment demonstrated safety and elicited immune responses, supported by the detection of serum antibodies (120).

### 8.5 PANVAC™

PANVAC™, a vaccine combining two viral vectors (vaccinia and fowlpox [FPV]) encoding MUC1 and CEA, administered with TRICOM, was evaluated in a randomized phase II trial. Thirty NMIBC patients with BCG-relapsed disease received subcutaneous PANVAC™ combined with BCG versus BCG alone. The regimen proved safe but showed comparable efficacy to BCG monotherapy (136). Subsequent development focused on CV301, a second-generation PANVAC comprising Modified Vaccinia Ankara (MVA) for priming and FPV for boosting, encoding CEA, MUC1, and TRICOM. A phase I trial combining CV301 with ICI suggested potential activity (137). However, a phase II study assessed subcutaneous CV301 combined with intravenous atezolizumab in 43 advanced UC cases refractory to or unfit for cisplatin therapy was terminated early during interim analysis due to insufficient efficacy (127).

### 8.6 Nadofaragene firadenovec

The recombinant immune modulator interferon α-2b (INFα-2b), shown to induce bladder tumor regression in preclinical studies (138, 139), was clinically explored in BCG-failure or BCG-unresponsive NMIBC patients through varying dosing regimens. These trials demonstrated tolerability and hinted at improved recurrence-free survival (140). To optimize delivery, INFα-2b was formulated as nadofaragene firadenovec (rAd-INFα/Syn3), a replication-deficient adenoviral vector enabling localized urothelial INFα-2b production to drive tumor regression (140). A subsequent phase III trial administered intravesical nadofaragene firadenovec (single doses repeated at 3, 6, and 9 months if no high-grade recurrence occurred) to BCG-unresponsive NMIBC patients. Results showed 53.4% achieving complete response (macroscopic tumor resolution) at 3 months, with 45.5% recurrence-free at 12 months. Notably, 91.9% of patients receiving ≥1 dose survived at 24 months, while only 2% experienced significant adverse events (141). As the first FDA-approved gene therapy for NMIBC, nadofaragene firadenovec emerges as a viable alternative for BCG-refractory patients (particularly those unfit for radical cystectomy) and may hold potential as a first-line option pending further confirmatory data (142).

## 9 Nucleic acid-based vaccines

Nucleic acid-based vaccines (DNA/mRNA) deliver genetic instructions encoding pathogen-specific antigens into host cells. The cells produce these antigens, triggering both antibody production and cellular immunity. Advantages include rapid design (no live pathogen handling), scalable manufacturing, strong immune activation (T-cells and antibodies), and stability (DNA resists refrigeration; mRNA avoids nuclear entry). mRNA vaccines also enable quick updates against variants. Both mRNA and DNA vaccines have been developed.

### 9.1 mRNA-based vaccines

mRNA vaccines are synthesized from exogenous plasmids and translated directly in the cytoplasm to produce peptides. Adjuvant co-administration enhances humoral immunity (143), while mRNA’s inherent immunogenicity further amplifies responses (144). Despite challenges like instability and delivery limitations, mRNA vaccines are efficient, safe (no genome integration risk), and scalable (145). Once the target sequence is identified, rapid mass production is achievable. The mRNA is ultimately degraded through natural cellular pathways (145). Non-replicating mRNA and self-amplifying RNA (SAM) constitute the two mRNA vaccine categories. Non-replicating mRNA contains: a 7-methylguanosine 5′ cap, 5′ untranslated region, open reading frame (OFR), 3′ untranslated region, and 3′ poly(A) tail (146, 147). SAMs incorporate dual-function OFRs, one encoding TAAs, the other harboring replication-essential genes for viral amplification, thereby generating multiple TAA copies (148). Protamine complexes with both substances, enabling TLR binding and heightened immune responses (81, 149).

Both SAM-based and non-replicating mRNA vaccines are synthesized via *in vitro* transcription using bacteriophage RNA polymerase and a DNA template (150). Strategies to enhance efficacy include optimizing mRNA stability [e. g., adjusting GC content, modifying nucleotides (151), resolving hairpin loops, replacing rare codons (152)] and improving delivery through innovations like encapsulating mRNA into positively charged liponanoparticles (77, 153). This liponanoparticles platform not only protects mRNA but also enables targeted cellular delivery, enhancing translation and reducing degradation (154). Additional advancements involve engineering the 5’ cap to recruit translation factors (155)and refining poly(A) tail sequences for stability and efficient translation initiation (156). Combined with epigenomic RNA modifications (157), these approaches aim to boost immunogenicity and therapeutic precision.

A personalized mRNA-4157 vaccine, lipid-encapsulated and encoding as many as 34 neoantigens, underwent assessment in a phase I dose-escalation trial for solid tumors. When administered alone as adjuvant treatment (*n* = 16) or alongside pembrolizumab (*n* = 63) in advanced disease, it produced no grade 3 adverse events. At 8-month median follow-up, 92% of adjuvant-only patients remained recurrence-free. In the combination cohort (including 6 UC patients), 3 CR and 2 PR were observed, demonstrating anti-tumor activity (154). A randomized phase II trial (N = 157) in resected high-risk melanoma showed adjuvant mRNA-4157 plus pembrolizumab prolonged RFS vs. pembrolizumab monotherapy (HR 0.561 [95% CI 0. 309–1.017]; *p* = .053). An excellent safety profile was observed: treatment-related AEs of grade ≥3 occurred in 25% of combination group patients versus 18% in the monotherapy group. No mRNA-4157-related grade 4–5 events occurred, and immune-mediated adverse event rates were comparable (16). These results prompted an ongoing phase III melanoma trial (NCT05933577) and a phase II trial (InterPath-005, NCT06305767) testing the combination in high-risk resected UC. The personalized neoantigen platform shows preliminary promise.

### 9.2 DNA-based vaccines

DNA-based vaccines utilize engineered bacterial plasmids encoding TAAs (158). Compared to mRNA, they exhibit greater stability and durability *in vivo*. The plasmid must enter the cell nucleus for transcription and translation, with a single plasmid generating multiple mRNA copies and peptides. Antigen presentation occurs either via MHC-I in somatic cells or through released peptides processed by APCs. The double-stranded DNA structure further enhances innate immunity (159). Intradermal delivery may enable direct DNA uptake by APCs, activating both humoral and cellular immune responses (160). Additionally, DNA vaccines can stimulate signaling pathways to induce GM-CSF/cytokine production (77) or be modified with immune agonists (e.g., CpG motifs, polymers, liposomes) (161). Nano-carriers (lipid-, protein-, polymer-, or inorganic-based) protect DNA from degradation (162). While selective and safe, a theoretical concern remains regarding rare genomic integration and potential oncogene activation (163).

To date, UC-focused clinical trials of DNA-based vaccines are undocumented. We present key preclinical progress, demonstrating that TGF-beta-encoding antisense oligonucleotide therapy could potentially arrest tumor cell proliferation *in vitro* and in UC mouse models (164). Combining BCG DNA-based and murine IL-12-based vaccines also demonstrated promising preclinical activity (165). The association of BCG DNA-based and murine IL-12based vaccines showed promising preclinical activity as well (81, 166).

Histone deacetylases (HDACs) remove acetyl groups from histones, leading to transcriptional suppression. Combining HDAC inhibitors with DNA-based vaccines may enhance efficacy. For instance, pairing an HDAC inhibitor with a CMV promoter-driven HER-2 DNA vaccine boosted lymphocyte infiltration and specific CTL activity, improving antitumor effects (167). Evaluation in UC mouse models of a DNA-based vaccine encoding Flk-1’s extracellular domain and complement 3d revealed tumor growth suppression and extended survival (168). Separately, in UC models, combining a tumor cell vaccine secreting IL-2, IL-4, and GM-CSF with an anti-HER-2 DNA vaccine enhanced antitumor activity, accompanied by elevated CD4+ T cells and anti-HER-2 antibodies (169).

## 10 Discussion

By reviewing the current research on the vaccine therapy for BCa, we have identified the potential mechanisms of action and characters of BCG, Nano BCG, Recombinant BCG, peptide-based BCG, cell-based vaccines, viral-based vaccines and nucleic acid-based vaccines in this field. These recent research findings include: BCG, as the most mature vaccine therapy for BCa, bind bladder tumor cells via fibronectin, inducing direct cytotoxicity and activating innate immunity through pathogen-recognition receptors. Nano BCG and recombinant BCG provide solutions to the limitations of traditional BCG therapy by enhancing the permeability and adhesion of BCG in bladder epithelium through nanotechnology and genetic engineering, inducing trained immunity and reducing the side effects associated with BCG. But carrier materials may trigger systemic inflammation or organ toxicity, with long-term biocompatibility remaining unverified. Another critical issue involves identifying exogenous antigen genes that can be optimally combined and introduced into BCG without cross-interference. High production costs and batch variability impede standardization and large-scale clinical adoption. Peptide-based vaccines utilize TAAs or TSAs, bounding MHC molecules to active CD8+/CD4+ T cell responses. By using carrier proteins or adding adjuvants, we can address the issues of MHC restriction of epitope recognition and its applicability in different populations. Clinical trials of peptide vaccines targeting BCa patients have shown positive efficacy, including improved survival rates and reduced tumor recurrence rates. Cell-based vaccines use the patient’s own or modified cells as antigen carriers or immune activation platforms to induce specific immune responses antigens (TAAs) through different strategies, break the tumor immune tolerance, activate T cells to recognize and kill tumor cells. Cell-based vaccines can be personalized for patients, targeting multiple TAAs simultaneously, reducing tumor immune evasion. They have shown potential in clinical trials. Viral-based vaccines use genetically engineered viruses (such as adenovirus, vaccinia virus, and coxsackievirus) to carry TAA encoding genes to infect host cells, releasing antigens through viral replication and activating immune responses. Viral vectors are diverse, each with its own advantages. The efficiency of antigen delivery is high, capable of activating cellular immunity and humoral immunity. Nucleic acid-based vaccines deliver genetic material (DNA or mRNA) encoding TAAs or neoantigens into host cells. These vaccines exploit the host’s cellular machinery to produce antigens, triggering both humoral and cellular immune responses. mRNA vaccines can encode patient-specific neoantigens (e.g., mRNA-4157) (154) for tailored immune targeting. DNA vaccines demonstrate better stability and durability compared to mRNA vaccines, while remaining preclinical for UC but demonstrate potent antitumor activity in murine models (e.g., HER-2/Flk-1 vaccines suppressing tumor growth) (167, 168).

For these newly emerging vaccine therapies that are in the clinical trial stage, we have identified several limiting factors that hinder research progress. First, the sample size is often insufficient. The subtypes of BCa are numerous, and the stratification of patients is complex, leading to limitations in the sample size of single-center trials. Second, there are significant differences in patient age, comorbidities, and past treatments (such as BCG exposure), which affect the efficacy evaluation. For example, elderly patients (especially those aged 50-70) may experience immune senescence that weakens the vaccine response, while those with no response to BCG need to be studied separately. Third, disputes over endpoint selection. The correlation between surrogate endpoints (such as immune response rate) and clinical endpoints (OS, PFS) is unclear. Fourth, manufacturing Complexity. Personalized vaccines (such as mRNA-4157) require custom production, which has long cycles and high costs. Due to the poor stability of the vaccines, cold chain transport further limits application in resource-scarce areas. Moreover, insufficient investment in research and development and lack of interdisciplinary collaboration, causing the disconnection between basic research and clinical translation.

While this review synthesizes current advancements in BCa vaccine therapies, several inherent limitations warrant acknowledgment. Primarily, as a descriptive review, it is susceptible to selection bias, potentially overlooking niche studies or unpublished negative data. The rapid evolution of this field also means that some very recent preclinical findings or newly initiated clinical trials may not be captured. Furthermore, the review does not provide a quantitative meta-analysis of efficacy or safety data across different vaccine platforms, which would offer a higher level of evidence for comparative effectiveness. The assessment of manufacturing complexities and cost barriers, while highlighted, is largely qualitative and could benefit from more detailed economic analyses.

Despite these limitations, this comprehensive overview holds significant clinical relevance. It provides clinicians and researchers with a structured framework for understanding the diverse vaccine strategies, their distinct mechanisms of action, and their respective developmental stages. By explicitly outlining the key challenges hindering clinical translation, including patient stratification difficulties, endpoint controversies, manufacturing hurdles, and immunosuppressive microenvironment, the review serves as a crucial guide for designing future clinical trials. It underscores the urgent need for standardized biomarker development to better predict responders, validates the exploration of combination therapy (e.g., vaccines with checkpoint inhibitors or novel adjuvants), and strongly advocates for increased collaborative efforts between immunologists, geneticists, bioengineers, and clinical oncologists. Critically, the review emphasizes the necessity of tailoring vaccine strategies to specific patient subgroups and highlights the practical realities of cost and logistics that will inevitably influence real-world clinical adoption. This synthesis thus not only informs ongoing research priorities but also equips clinicians with the knowledge to critically evaluate emerging vaccine therapies and anticipate their potential integration into future BCa treatment paradigms.

The dawn of cancer vaccine therapies for BCa has gradually illuminated the path from preclinical research to clinical application. However, different types of vaccines inherently possess their own unavoidable side effects and limitations. Addressing these challenges through emerging technologies such as nanotechnology and genetic engineering, or via strategic combination with immunoadjuvants to synergize therapeutic advantages while mitigating shortcomings, remains a prolonged journey requiring innovative exploration. Future research should prioritize mechanistic investigations of immune responses, particularly through in-depth analysis of the immunological mechanisms underlying clinically established intravesical BCG immunotherapy. Concurrently, critical efforts should focus on resolving key challenges including the immunosuppressive TME and limitations in antigen selection, which are pivotal for advancing this therapeutic paradigm. The remarkable progress in BCa vaccine development heralds a transformative era where tailored immunotherapies could soon turn aggressive tumors into preventable adversaries, offering renewed hope for lasting cures.

## 11 Conclusion

In conclusion, this article reviews the existing research on vaccine therapy for BCa tumors, follows the research on the mechanism of BCG intravesical immunotherapy, revealing its limitations, and summarizes the clinical/preclinical studies on BCG vaccines optimized based on nanotechnology and genetic engineering, alongside other vaccine types for the treatment of BCa tumors. Building on these analyses, this article reflects on the current challenges and future research directions in the field. It is hoped that this study will provide effective assistance in better understanding vaccine therapy for BCa tumors and inspire innovative approaches in cancer vaccine development for BCa tumors.
